# *Chlamydia pneumoniae* is present in the dental plaque of periodontitis patients and stimulates an inflammatory response in gingival epithelial cells

**DOI:** 10.15698/mic2019.04.674

**Published:** 2019-03-11

**Authors:** Cássio Luiz Coutinho Almeida-da-Silva, Tamer Alpagot, Ye Zhu, Sonho Sierra Lee, Brian P. Roberts, Shu-Chen Hung, Norina Tang, David M. Ojcius

**Affiliations:** 1 Department of Biomedical Sciences, Arthur A. Dugoni School of Dentistry, University of the Pacific, San Francisco, CA 94103, USA.; 2 Department of Periodontics, Arthur A. Dugoni School of Dentistry, University of the Pacific, San Francisco, CA 94103, USA.; 3 College of Letters and Science, University of California, Berkeley, CA 94720, USA.; 4 Program of Doctor of Dental Surgery, Arthur A. Dugoni School of Dentistry, University of the Pacific, San Francisco, CA 94103, USA.; 5 College of Letters and Science, University of California, Los Angeles, CA 90095, USA.

**Keywords:** Chlamydia, inflammasome, periodontitis, inflammation, caspase-1

## Abstract

*Chlamydia pneumoniae* is an airborne, Gram-negative, obligate intracellular bacterium which causes human respiratory infections and has been associated with atherosclerosis. Because individuals with periodontitis are at greater risk for atherosclerosis as well as respiratory infections, we in-vestigated the role of *C. pneumoniae* in inflammation and periodontal dis-ease. We found that *C. pneumoniae* was more frequently found in subgingival dental plaque obtained from periodontally diseased sites of the mouth versus healthy sites. The known periodontal pathogens, *Porphyromonas gingivalis* and *Aggregatibacter actinomycetemcomitans*, were also found in the plaque. In addition, *C. pneumoniae* could efficiently invade human gingival epithelial cells (GECs) *in vitro*, causing translocation of NF-κB to the nucleus along with increased secretion of mature IL-1β cytokine. Supernatants collected from *C. pneumoniae*-infected GECs showed increased activation of caspase-1 protein, which was significantly reduced when *nlrp3* gene expression was silenced using shRNA lentiviral vectors. Our results demonstrate that *C. pneumoniae* was found in higher levels in periodontitis patients compared to control pa-tients. Additionally, *C. pneumoniae* could infect GECs, leading to inflammation caused by activation of NF-κB and the NLRP3 inflammasome. We propose that the presence of *C. pneumoniae* in subgingival dental plaque may contribute to periodontal disease and could be used as a potential risk indicator of perio-dontal disease.

## INTRODUCTION

Periodontitis is the most common oral inflammatory disease [1]. It involves inflammation and destruction of the attachment apparatus of the teeth (i.e., gingiva, periodontal ligament, root cementum and alveolar bone). Periodontitis begins with an infection of the gingival tissue by etiologic agents. Bacterial invasion of the gingiva elicits a host immune response which begins with inflammation of the gingiva, progressing to destruction of deep periodontal tissues and loss of alveolar bone. Even though over 600 species of bacteria are present in the human oral microbiome, only about ten have been identified as putative pathogens causing periodontal disease [2]. These putative pathogens are mainly Gram-negative anaerobic bacteria and include *Aggregatibacter actinomycetemcomitans*, *Porphyromonas gingivalis, Porphyromonas intermedia, Bacteroides forsythus, Campylobacter rectus, Eubacterium nodatum, Porphyromonas micros, Staphylococcus intermedius and Treponema spp*. [3]. Of these ten species, *A. actinomycetemcomitans, P. gingivalis* and *B. forsythus* are most commonly associated with periodontitis [4–6].

Chlamydiae are obligate, intracellular bacteria that have a biphasic developmental cycle [7–9]. In the early stages, infectious forms called elementary bodies (EBs) enter mucosal cells and reside in membrane-bound vacuoles called inclusions. Within a few hours, the EBs differentiate into larger, metabolically active reticulate bodies (RBs) which are non-infectious. After several rounds of replication, RBs differentiate back into EBs and are released from the host cell, ready to infect neighboring cells.

The cells infected by *Chlamydia* include epithelial cells, smooth muscle cells, fibroblasts, osteoblasts, monocytes/macrophages and dendritic cells [10–13]. These susceptible host cells recognize chlamydial antigens by several means: through cell surface receptors, endosomal receptors, and cytosolic innate immune sensors [10]. Activation of these receptors leads to the release of pro-inflammatory cytokines and chemokines which recruit inflammatory cells to eliminate the infectious agent. Under immunopathologic conditions, such as with chronic immune activation, these events can lead to tissue damage and scarring.

The two major chlamydial species that are pathogenic to humans are *Chlamydia trachomatis* and *Chlamydia pneumoniae* [14]. Infection with *C. trachomatis* can lead to non-congenital blindness and genital tract infections and complications such as pelvic inflammatory disease, infertility, ectopic pregnancy, urethritis and cervical cancer. *C. pneumoniae* causes respiratory infections including pneumonia, bronchitis, pharyngitis and sinusitis. Approximately 10% of community-acquired pneumonia cases are caused by *C. pneumoniae* infection [15]. *C. pneumoniae* has also been linked to asthma, arthritis, atherosclerosis, stroke, multiple sclerosis and Alzheimer's disease [12, 15–18]. Chlamydial infections are treatable with antibiotics, but no vaccines are available for prevention.

*C. pneumoniae* has not been traditionally considered a member of the human oral microbiome and was not thought to infect oral cells [2, 19, 20]. However, Rizzo *et al.* reported infecting human gingival fibroblasts *in vitro* and eliciting secretion of the cytokines, interleukin-6 and interleukin-10 [21, 22]. In another study, *C. pneumoniae* was found in the gingival crevicular fluid of one periodontitis patient with characteristics similar to a clinical strain [23]. Moreover, a characterization of subgingival dental plaque revealed that *C. pneumoniae* was present in one out of twelve clinical samples obtained from periodontitis patients [24]. Unfortunately, in these studies, the clinical sample sizes were small and no comparisons to healthy patient controls were made. In addition, no demonstration had been made that *C. pneumoniae* can invade the oral epithelium, one of the first and major barriers to oral disease. Currently, it is still not known whether *C. pneumoniae* infects gingival epithelium and subsequently promotes a host-mediated immune response that leads to periodontitis.

In this study, using 80 dental plaque samples, we examined whether *C. pneumoniae* can be found in subgingival dental plaque and whether its presence correlated with periodontal disease. In addition, we examined the ability of *C. pneumoniae* to invade gingival epithelial cells and elicit a host inflammatory response.

## RESULTS

### *C. pneumoniae* is more frequent in subgingival plaque from patients with periodontal disease than in healthy controls

Eighty subgingival dental plaque samples were obtained from 20 subjects with and without periodontal disease. In the healthy group, subgingival plaque samples were obtained from four periodontally “healthy sites”. In patients with periodontitis, subgingival plaque samples were obtained from both “healthy sites” and “periodontitis sites” (two healthy sites and two diseased sites per periodontitis patient). The demographic characteristics of the studied population are presented in Table 1; no statistically significant differences were observed among groups. Table 2 shows the clinical characteristics for our study population. In the periodontitis group, diseased sites exhibited generalized moderate chronic periodontitis.

**TABLE 1.** Demographic characteristics (mean and SD) of subjects in the Healthy and Disease groups (N = 20).

Variable		Healthy Group (n = 10)	Disease Group (n = 10)
**Age**		36.9 + 10.8 years	40.5 + 11.6 years
**Sex**	Female	6	4
	Male	4	6
**Race**	White	6	3
	Asian	3	2
	Hispanic	1	4
	African American	0	1
**Education Level**	High School	2	5
	Colllege/Postgraduate	8	5

* Individuals with current or past history of smoking were not included in the study. No statistical difference was observed between the two groups.

**TABLE 2.** Clinical measurements of healthy and diseased sites.

	Healthy Sites (n = 60)	Diseased Sites (n = 20)
**Plaque Index**	0.51 ± 0.23	1.83 ± 0.71
**Gingival Index**	0	1.44 ± 0.32
**Probing Depth (mm)**	1.5 ± 0.17	5.75 ± 0.97
**Attachment Loss (mm)**	0.5 ± 0.11	4.25 ±1.71

**Figure 1:**
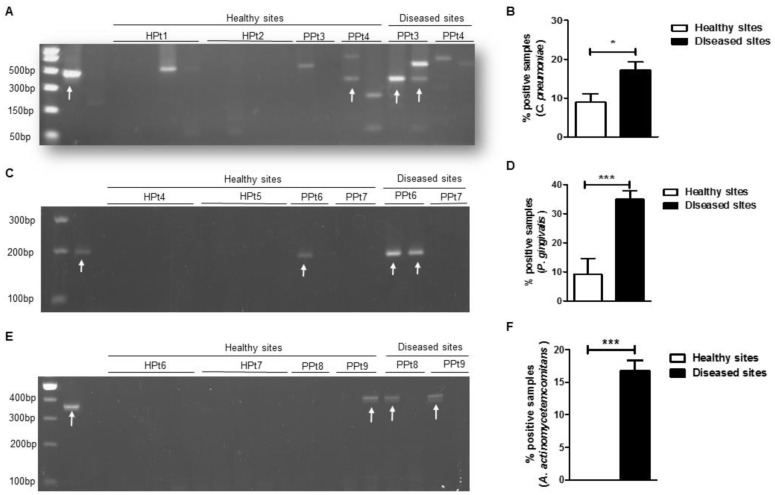
FIGURE 1: *C. pneumoniae* is present more frequently in subgingival plaque obtained from periodontally diseased sites vs. healthy sites. Dental plaques were obtained from subgingival locations in ten healthy patients (HPt, four sites for each one) and in ten patients with periodontal disease (PPt). For patients with periodontal disease, subgingival dental plaques were obtained from both areas with periodontitis (P, two sites per subject) and areas without periodontitis (H, two sites per subject). All dental plaque samples were subjected to PCR followed by agarose gel electrophoresis. **(A, C, E)** Representative gel electrophoretogram of PCR products screening *C. pneumoniae* **(A)**, *P. gingivalis* **(C)** and *A. actinomycetemcomitans* **(E)**. Quantification of *C. pneumoniae* **(B)**, *P. gingivalis* **(D)** and *A. actinomycetemcomitans* **(F)** 16S rRNA, PCR products. **(B, D, F)** Healthy sites: n = 40 (only from healthy donors) and diseased sites: n = 20. For each dental plaque sample, seven different runs were performed for *C. pneumoniae* and three different runs for *P. gingivalis* and *A. actinomycetemcomitans.* Arrows indicate positive bands in the expected bp size. Error bars represent ± SD; 2-sided, paired Student's *t* test, *P ≤ 0.05, ***P≤0.001.

After extracting total nucleic acids from the dental plaque samples, conventional PCR was performed to amplify the gene encoding *C. pneumoniae* 16S rRNA and the resultant PCR products were separated on an agarose gel (**Fig. 1A**). In our sample population, we found that *C. pneumoniae* was significantly more frequent in the diseased group compared with the healthy group (**Fig. 1B**). Because *C. trachomatis* was previously reported in the oral cavity of patients with periodontitis [25], we tested the specificity of our primers using control samples of *C. pneumoniae* and *C. trachomatis* and found that our *C. pneumoniae* 16s rRNA primer was specific only for the *C. pneumoniae* species (Supplementary Figure S1). We also screened the same samples for two well-known periodontal pathogens: *P. gingivalis* and *A. actinomycetemcomitans* [3]. As expected, we detected significantly higher levels of *P. gingivalis* (**Fig. 1C** and **D**) and *A. actinomycetemcomitans* (**Fig. 1E** and **F**) 16S rRNA gene by PCR in diseased samples compared with the control groups. Therefore, in our sample population, the presence of *C. pneumoniae, P. gingivalis* and *A. actinomycetemcomitans* in dental plaque from periodontally diseased sites was significantly more common than in healthy sites.

### *C. pneumoniae* infects human gingival epithelial cells *in vitro*

**Figure 2:**
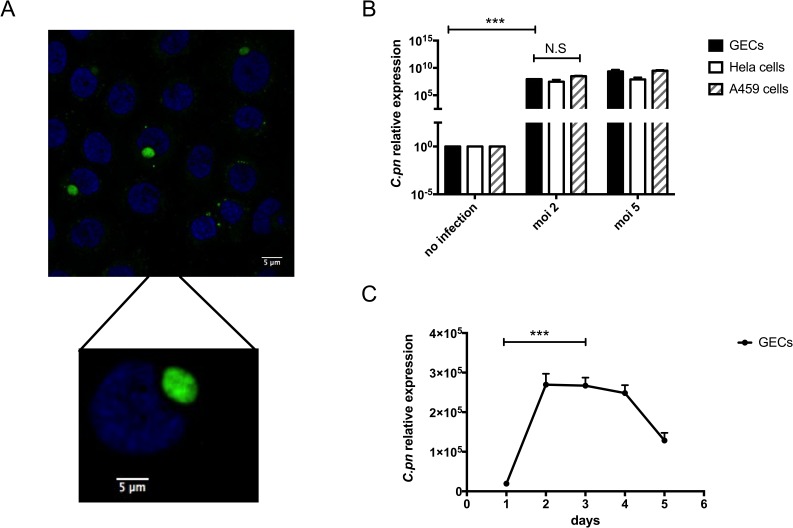
FIGURE 2: *C. pneumoniae* infects human gingival epithelial cells. **(A)**. Gingival epithelial cells (GECs), infected four days with *C. pneumoniae* at MOI of 5, were incubated with a FITC-conjugated mAb against *Chlamydia* (green) for 15 minutes, and the nuclei were counterstained with Hoechst (blue). **(B)** qPCR of hGECs, HeLa cells, and A549 cells infected with *C. pneumoniae* for two days at MOI of 0, 2 and 5. qPCR assays were performed in triplicate, n = 4 independent experiments. Error bars represent ± SD; 2-sided, paired Student's *t* test, ***P < 0.001. (C) GECs were infected with *C. pneumoniae* at MOI of 5 and infected samples were collected 1, 2, 3, 4 and 5 days post-infection for qPCR analysis. Assays were performed in triplicates, n = 5 independent experiments. Error bars represent ± SD; 2-sided, paired Student's *t* test, ***P < 0.001.

Since our clinical data showed higher levels of *C. pneumoniae* in dental plaque from periodontal disease patients compared with controls, we next investigated a possible mechanism that could contribute to development of inflammation and periodontitis due to *C. pneumoniae*. Thus, we first determined whether *C. pneumoniae* can infect human gingival epithelial cells (GECs). These cells were chosen because they are in intimate contact with bacteria present in subgingival plaques and they represent the first barrier against foreign invasion in the oral cavity. **Figure 2A** shows GECs that were incubated with *C. pneumoniae* at a MOI (multiplicities of infection) of 5 for 4 days. By immunofluorescence microscopy, the fluorescein isothiocyanate-stained bacterial RBs can be clearly observed inside the inclusions within the epithelial cells.

*C. pneumoniae* is known to infect human epithelial cells from different tissue types with varying degrees of efficiency. These different tissue types include the lung, larynx and cervix [26–28]. In **Figure 2B**, the efficiency of *C. pneumoniae* infection of GECs was compared with infection of A549 human lung epithelial cells and HeLa human cervical epithelial cells. Using different MOI, *C. pneumoniae* could invade and replicate in GECs to a comparable extent as in the cells most often used to study chlamydial infection: HeLa cells and A549 cells. While examining the kinetics of *C. pneumoniae* infection of GECs, peak levels of infection were seen between days 2 and 4 post-infection. After day 4, intracellular *C. pneumoniae* levels began to drop. These kinetics are similar to what had been previously reported for the infection of HeLa cells by *C. pneumoniae* [29].

### *C. pneumoniae* infection of GECs causes activation of NF-κB

**Figure 3:**
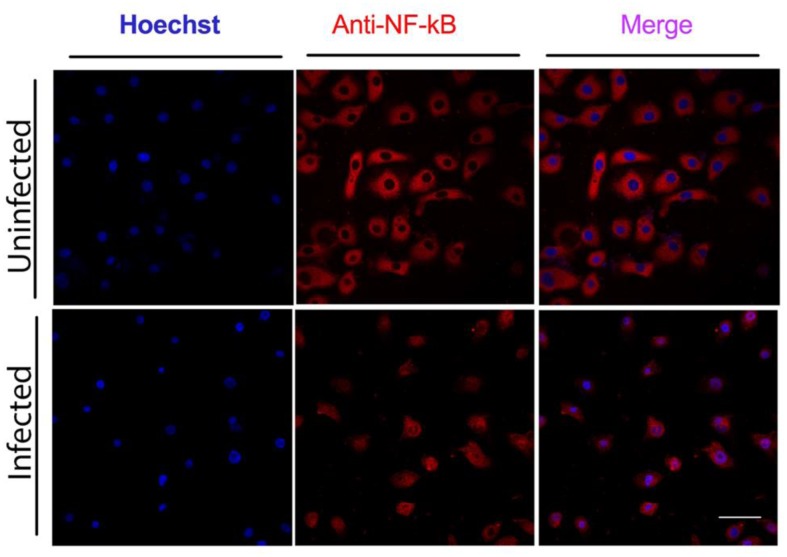
FIGURE 3: Activation of NF-κB in hGECs infected with *C. pneumoniae*. hGECs were infected with *C. pneumoniae* at a MOI of 5 for three days and immunocytochemistry performed using a NF-κB/p65 monoclonal antibody (red). hGEC cell nuclei were counterstained with Hoechst (blue).

The pro-inflammatory transcription factor NF-κB is one of the key regulators of genes involved in the immune and inflammatory response. Therefore, we looked at its activation in GECs infected with *C. pneumoniae*. GECs incubated with *C. pneumoniae* for three days at a MOI of 5 showed nuclear NF-κB/p65 staining, compared with uninfected cells, where NF-κB was in the cytosol (**Fig. 3**). The translocation of NF-κB/p65 from the cytosol to the nucleus indicates activation of this transcription factor.

### Infected GECs secrete IL-1β and activated caspase-1

**Figure 4:**
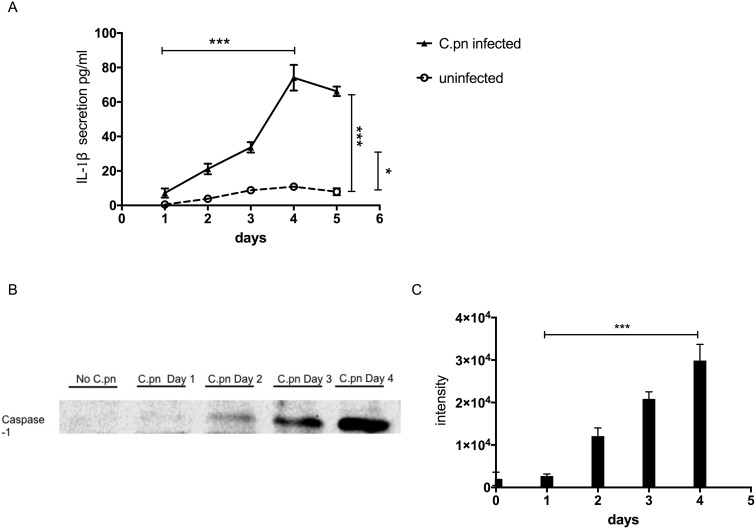
FIGURE 4: *C. pneumoniae* infection activates caspase-1 and IL-1β release. Equal numbers of GECs were infected with *C. pneumoniae* and cultured in equal volumes of medium for five days in **(A)** and four days in **(B, C)** at a MOI of 5. At 0, 1, 2, 3, 4 and 5 days post-infection, supernatants were collected and analyzed for the presence of mature IL-1β or activated caspase-1 protein. **(A)** IL-1β ELISA. Assays were performed in triplicates, n = 3 independent experiments. **(B)** Western blot representative gel and **(C)** quantification of active caspase-1 proteins. Three independent experiments were performed. For panels A and C: error bars represent ± SD; 2-sided, paired Student's *t* test, ***P < 0.001.

One of the genes turned on by NF-κB is *il-1*β (interleukin-1β). The *il-1*β gene is first expressed as a precursor protein, pro-IL-1β, which is subsequently cleaved by the protease caspase-1 to become the mature form, IL-1β protein. Secreted IL-1β promotes and amplifies the inflammatory response. We therefore examined whether activation of NF-κB in GECs infected by *C. pneumoniae* also results in increased secretion of IL-1β cytokine. Over a period of 1 – 5 days, we found that IL-1β secretion increased steadily over time, peaking at 4 days post-infection (**Fig. 4A**). After four days, secreted IL-1β protein began to decline, correlating approximately with the time course of *C. pneumoniae* infection. Not surprisingly, the kinetics of IL-1β secretion also paralleled the kinetics of caspase-1 activation. **Figures 4B** and **4C** show that the amount of 22 kDa active form of caspase-1 in cellular supernatants of infected GECs increased over time from 0 to 4 days post-incubation.

### The NLRP3 inflammasome participates in caspase-1 activation

NF-κB activation also results in upregulation of *nlrp3* (NOD-like receptor family, pyrin domain containing 3; also known as cold induced autoinflammatory syndrome 1, *cias1*). The NLRP3 protein is a member of the nucleotide-binding oligomerization domain (NOD), leucine-rich repeat (LRR)-containing protein family (NLR family). Other members of the NLR family include NLRP1, NLRC4, AIM2 and pyrin. Together with the apoptosis-associated speck-like protein containing a CARD (ASC, also known as PYCARD) and pro-caspase-1, NLRs form the oligomeric signaling platform called inflammasome [30, 31]. One of the biochemical functions of the inflammasomes is to activate caspase-1.

**Figure 5:**
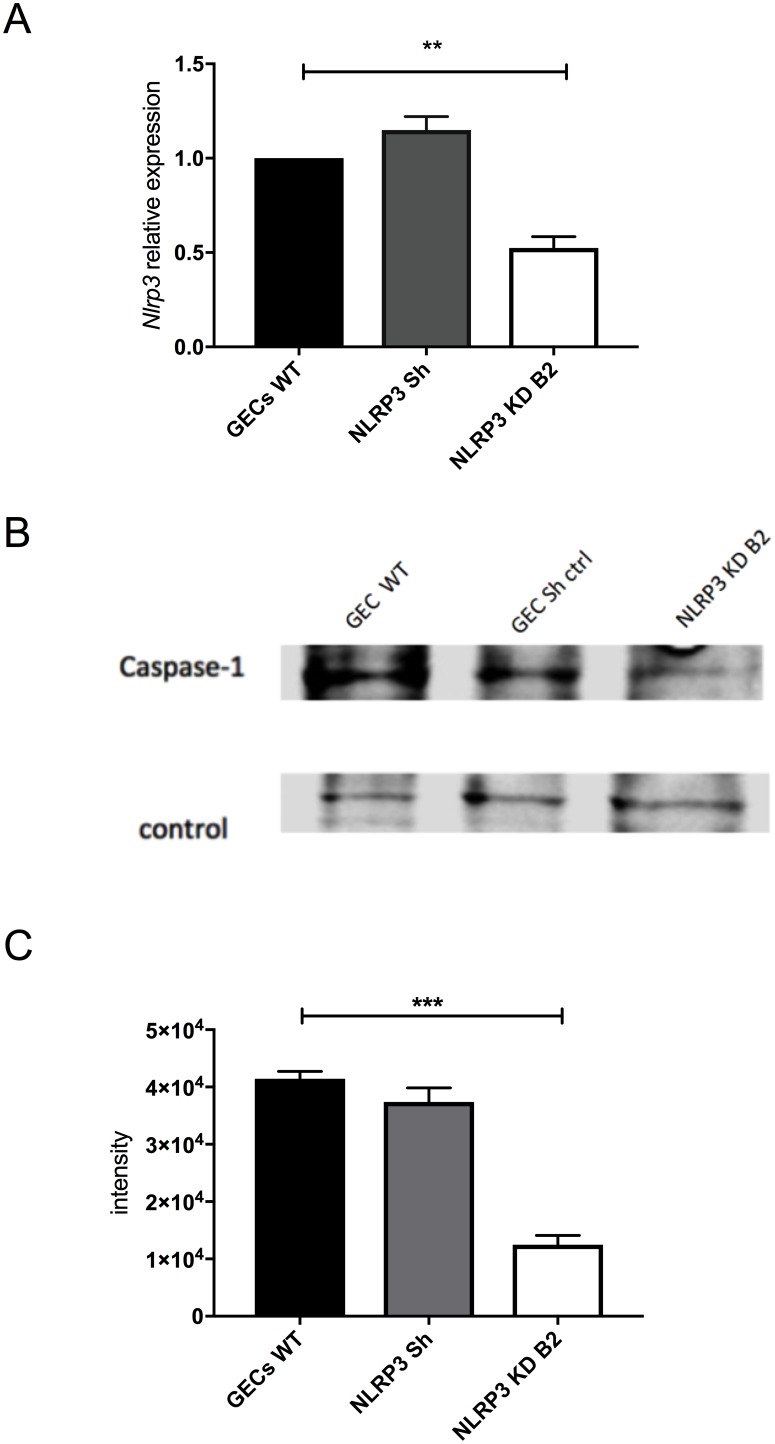
FIGURE 5: Inhibition of *nlrp3* gene expression reduces caspase-1 activation. **(A)** qPCR for *nlrp3* gene expression was performed using RNA extracted from GECs transduced with lentiviruses carrying shRNAs targeting *nlrp3* (*nlrp3* shRNA) or non-targeting control shRNAs (*ctl* shRNA). qPCR assays were performed in triplicates, n = 4 independent experiments. Error bars represent ± SD; 2-sided, paired Student's *t* test, **P < 0.01. **(B)** Western blot representative image and **(C)** quantification of caspase-1 of supernatants from lentivirus-transduced GECs. Three independent experiments were performed; a representative blot is shown. Error bars represent ± SD; 2-sided, paired Student's *t* test, ***P < 0.001.

Since *C. pneumoniae* infection of GECs caused activation of caspase-1 (**Fig. 4B**), we investigated if an inflammasome may be responsible for this activity. The NLRP3 inflammasome was viewed as a likely candidate since earlier studies showed that treatment with the danger signal, extracellular ATP, and infection with the periodontal pathogen *Fusobacterium nucleatum* could activate the NLRP3 inflammasome in GECs [32–36]. To determine whether the NLRP3 inflammasome was involved in caspase-1 activation during *C. pneumoniae* infection, we inhibited *nlrp3* expression in GECs using specific shRNAs packaged into lentiviral particles. **Figure 5A** shows that *nlrp3* mRNA expression in the lentivirus-transduced cells was roughly half of that seen in non-transduced cells or cells transduced with irrelevant shRNA control. The decrease in *nlrp3* gene expression in GECs also showed a corresponding decrease in caspase-1 activation during infection, as seen in **Figure 5B and C**. The data therefore indicate that the NLRP3 inflammasome is involved in the proteolytic cleavage and activation of caspase-1 during *C. pneumoniae* infection of GECs.

## DISCUSSION

The human oral microbiome consists of over 600 abundant species of bacteria, of which fewer than a dozen have been associated with periodontal disease [2, 37, 38]. Although there are recent data to suggest *C. pneumoniae* may be part of the oral microbiota and may play a role in periodontitis, the data are scant and incomplete. Moreover, *C. pneumoniae* has never been shown to interact with the gingival epithelium. In the present study, we studied a large number of clinical samples along with healthy patient controls and used human GECs as a relevant *in vitro* model to investigate *C. pneumoniae* infection of oral tissue.

In this study we used 80 subgingival dental plaques obtained from patients with and without periodontitis and showed higher levels of *C. pneumoniae* in diseased sites compared with control sites. *P. gingivalis* and *A. actinomycetemcomitans*, two well-known periodontopathogens, were also found in higher levels in diseased sites compared to controls. To the best of our knowledge, this is the first time that *C. pneumoniae* and other periodontopathogens are found in the same dental plaque samples. Moreover, it is not known how or whether *C. pneumoniae* can contribute to the development of periodontitis. In this context, our study showed for the first time the presence of *C. pneumoniae* in dental plaques more often in periodontitis samples and in healthy sites, comparing healthy and diseased groups (80 sites from 20 patients). To confirm our initial observations, future studies will use a larger sample population in order to establish a correlation between the disease and the presence of *C. pneumoniae*; analyze IgG and IgM antibodies against *C. pneumoniae* in blood samples from the same population of patients; and examine whether viable *C. pneumoniae* can be recovered from dental plaque samples. Additionally, we did not observe differences between the groups regarding sex, race and education levels (Table 1), probably because of our small size population.

In this study, we used human GECs as an *in vitro* model to examine *C. pneumoniae* invasion of the oral epithelium and induction of host inflammation. We found that *C. pneumoniae* can infect human GECs, with maximal growth and replication within inclusions inside the host after four days post-incubation. This developmental growth pattern is similar to what has been reported for infection of cervical epithelial (HeLa) cells [29], which until now have been the main cell type used for studies of *C. pneumoniae* infection. Our findings that *C. pneumoniae* infection caused NF-κB nuclear translocation and upregulation of IL-1β in GECs, in large part through the involvement of the NLRP3 inflammasome and subsequent activation of caspase-1, are also consistent with findings using other cell types. For instance, *C. pneumoniae* infection of smooth muscle cells, monocytes/macrophages, dendritic cells, as well as epithelial cells from non-oral tissues has been reported to result in NF-κB activation and upregulation of proinflammatory cytokines, including IL-1β [39–43]. Activation of the NLRP3 inflammasome and caspase-1 has been documented in macrophages for *C. pneumoniae* [44, 45] and in cervical epithelial cells for *C. trachomatis* [46].

IL-1β is a major player in the periodontal inflammatory process underlying chronic periodontitis [47]. In addition, oral pathogens are known to activate NF-κB in epithelial cells and macrophages [48]; and periodontopathogens such as *P. gingivalis* and *F. nucleatum* activate NF-κB in epithelial cells and increase expression of proinflammatory gene transcripts [35, 36, 49, 50]. Thus, we hypothesize that, upon *C. pneumoniae* infection, epithelial cells, macrophages and fibroblasts in the periodontium may secrete pro-inflammatory cytokines involved in the pathogenesis of periodontal disease.

Although our results show that there are higher levels of *C. pneumoniae* in dental plaque samples with periodontal disease compared with healthy control groups, and the results with infected GECs suggest how *C. pneumoniae* could contribute to inflammation in the oral cavity, our findings on *C. pneumoniae* organisms in subgingival dental plaques of periodontitis patients do not prove that the bacteria cause disease or whether, instead, periodontal disease favors their accumulation in dental plaque. Considering that periodontitis is a polymicrobial disease caused by complex interactions between distinct pathogens [37, 38, 51], it is possible that other microbial agents present in the dental plaques of periodontitis patients could be the cause of disease, and *C. pneumoniae* could simply be modifying the composition of the microbial community in dental plaques. Future studies will need to examine the interaction of other pathogenic microorganisms in dental plaques with *C. pneumoniae* to determine how they elicit a host inflammatory response and subsequent development of periodontitis.

Moreover, it is not known whether the *C. pneumoniae* in subgingival dental plaques is infectious in the oral cavity; or whether *C. pneumoniae* in dental plaques could behave as a reservoir for the pathogen that could lead to lung infections. Additionally, the gingival epithelium has stratified squamous keratinized cells and most studies so far, including our own, analyzed chlamydial infection in monolayer cell models of infection. Even though *C. pneumoniae* has never been reported to be present in the human gingival epithelium, a recent study showing that *C. trachomatis* infects three-dimensional organotypic stratified squamous epithelial cultures [52] suggests that similar events may occur in the oral cavity. Finally, an oral reservoir of *C. pneumoniae* could infect circulating immune cells and thus contribute to other inflammatory diseases. This could conceivably be the case for atherosclerosis, since individuals with periodontitis are known to be at a higher risk for foam cell formation and arteriosclerosis [12, 53]. Clearly, more studies are needed to elucidate the role of *C. pneumoniae* in the mouth and systemic diseases, which should lead to a better understanding of the pathogenesis of periodontal disease in relation to systemic diseases in general.

## MATERIALS AND METHODS

### Clinical subjects and samples

The study population consisted of 20 medically healthy subjects with an average of 38.7 + 11.2 years (range 26.8 to 62.3) and included ten women and ten men. Ten subjects were in the disease group (two periodontitis sites and two healthy sites for each participant were the study sites collected), and ten subjects were in the periodontally healthy group (four healthy sites for each subject were the study sites). All study participants signed a consent form approved by the Institutional Review Board of University of the Pacific. Exclusion criteria for participants included the following: individuals who were younger than 25 years of age, who did not have at least two none adjacent periodontitis sites and two healthy sites in the disease group, and four healthy sites in the healthy group (excluding third molars), who did not have 20 teeth, who required premedication with antibiotics prior to periodontal exam, who had taken antibiotics or anti-inflammatory drugs within the last six months, or gone through periodontal therapy within the last six months, or who are current or past smokers.

### Study design

A single calibrated examiner performed all clinical measurements and plaque sampling. Clinical measurements including plaque index [54], gingival index [55], bleeding on probing, duplicate measurements of probing depth and attachment loss were recorded in 20 periodontitis sites and 60 healthy sites using a North Carolina probe.

The criteria for healthy sites and periodontitis sites were as follows. Healthy sites (including sites with gingival recession) were defined as: Gingival index = 0; probing depth < 4 mm; attachment loss < 3 mm. For periodontitis sites: Gingival index >0; probing depth > 4 mm; attachment loss > 3 mm.

### Plaque collection and qPCR analysis of clinical samples

Using sterile Gracey curettes, subgingival dental plaques were obtained from consenting patients, and stored in a -80°C freezer until the day of analysis. A total of 80 subgingival plaque samples were collected from the participants in diseased and healthy study groups.

The plaque from the curette was resuspended in 350 µl of RTF buffer (0.045% K_2_HPO_4_, 0.09% NaCl, 0.09% (NH_4_)_2_SO_4_, 0.018% MgSO_4_, 0.038% EDTA, 0.04% Na_2_CO_3_, 0.02% dithiothreitol) [56]. Samples were boiled at 96°C for 10 min and centrifuged, and supernatants were collected for nucleic acids analysis.

PCR was performed to amplify the 16S rRNA gene of *C. pneumoniae, C. trachomatis, P. gingivalis* and *A. actinomycetemcomitans* using the Fast Cycling PCR Kit (Qiagen, Valencia, CA, USA). A hot-start protocol was used at 95°C (5 min) and the PCR cycling parameters were: denaturation at 96°C (5 sec), annealing at 45°C (5 sec) for *C. pneumoniae,* 57°C (5 sec) for *C. trachomatis*, 52°C (5 sec) for *P. gingivalis* and *A. actinomycetemcomitans*, and extension at 68°C (15 sec) for *C. pneumoniae*, 68°C (9 sec) for *C. trachomatis* and 68°C (4 sec) for *P. gingivalis* and *A. actinomycetemcomitans* in a total of 40 cycles, followed by a final extension at 72°C (1 min). Primer sets, final concentration and amplicon size were as follows for the different pathogens: *C. pneumoniae* 16S rRNA Forward 5′-TGACAACTGTAGAAATACAGC-3′, *C. pneumoniae* 16S ribosomal RNA Reverse 5′-CGCCTCTCTCCTATAAAT-3′ [57], both primers were used at 0.4 µM final concentration resulting in a 465-bp amplicon; *C. trachomatis* 16S rRNA Forward 5′-GGCGTATTTGGGCATCCGAGTAACG-3′, *C. trachomatis* 16S rRNA Reverse 5′-TCAAATCCAGCGGGTATTAACCGCCT-3′ [58], both primers were used at 0.3 µM final concentration, resulting in a 315-bp amplicon; *P. gingivalis* 16S rRNA Forward 5′-TGTAGATGACTGATGGTGAAAACC-3′, *P. gingivalis* 16S rRNA Reverse 5′-ACGTCATCCCCACCTTCCTC-3′ [59], both primers were used at 0.3 µM final concentration, resulting in a 198-bp amplicon; *A. actinomycetemcomitans* 16S rRNA Forward 5′-ATTGGGGTTTAGCCCTGGTG-3′, *A. actinomycetemcomitans* 16S rRNA Reverse 5′-ACGTCATCCCCACCTTCCTC-3′ [59], both primers were used at 0.5 µM final concentration, resulting in a 360-bp amplicon. All amplicon sizes were examined by electrophoresis in a 2% agarose gel stained with a final concentration of 0.5 µg/ml ethidium bromide (EtBr; Invitrogen, Carlsbad, CA) and visualized under UV light. To be used as positive controls, genomic DNA was isolated from *C. pneumoniae* (CM-1, ATCC and ATCC VR-1360D, *Chlamydophila pneumoniae*, Strain CM-1), *P. gingivalis* (ATCC^®^ 33277), *A. actinomycetemcomitans* (ATCC^®^ 29522) using TRIzol (Thermo Fisher Scientific), and DNA of *C. trachomatis* was obtained from ATCC (ATCC^®^ VR-885D, Serovar D; Strain UW-3/Cx). Ultra-pure water was used as negative control indicating primers were specific without self-annealing detections.

The scoring of the electrophoretograms was done by two different individuals with double-blinded samples. Neither the status of the donor (healthy patient vs. periodontitis patient) nor the status of the subgingival location (healthy site vs. diseased site) were known to the scorers. Samples were scored as “positive” if the ethidium bromide-stained gel band corresponding to the different bacteria, at the expected size, was visible to the scorer's naked eye.

### Mammalian cell culture

Human immortalized gingival epithelial cells (GECs) were previously described [35]. GECs were cultured in 1X defined Keratinocyte Serum-Free Medium (KSFM: Gibco Life Technologies, Gaithersburg, MD, USA) supplemented with 30 µg/ml bovine pituitary extract (Gibco Life Technologies), and 0.2 ng/ml of human recombinant epidermal growth factor (Gibco Life technologies) at 37°C, 5% CO_2_.

Human cervical epithelial cells, HeLa, were obtained from ATCC^®^ (CCL-2™) and cultured in Minimum Essential Medium (Gibco Life Technologies) supplemented with 10% FBS (Invitrogen) at 37°C, 5% CO_2_, as previously described [46]. Human lung epithelial cells, A549, were obtained from ATCC^®^ (CCL-185™) and cultured in F-12 medium (Gibco Life Technologies) supplemented with 10% FBS at 37°C, 5% CO_2_.

### Infection of GECs with *C. pneumoniae*

*C. pneumoniae* (CM-1, ATCC, Manassa, VA) was grown and quantified as previously described [60, 61].

GECs were seeded on 18 mm glass cover slips (Fisher Scientific, Hampton, NH, USA) or 6-well plates (Corning, Corning, NY, USA) and grown to 60% confluency, after which *C. pneumoniae* bacteria were added at various multiplicity of infection (MOI) and incubated for various lengths of time as indicated in the figure legends.

### *C. pneumoniae* immunofluorescence

GECs were grown on 18 mm glass coverslips and infected with *C. pneumoniae* at a MOI of 5. After four days, supernatants were aspirated, and the *C. pneumoniae*–infected cells fixed with freezing cold methanol for 10 minutes. The cells were then washed three times with 1X phosphate-buffered saline (PBS), permeabilized with 0.1% Triton X-100 in PBS for 15 minutes and blocked with 5% bovine serum albumin (BSA: Sigma Aldrich St. Louis, MO, USA) in PBS for 1 hour. To visualize *C. pneumoniae* in the GECs, infected cells were incubated with a fluorescein isothiocyanate (FITC)-conjugated monoclonal antibody from the *IMAGEN*™*Chlamydia* Kit (Invitrogen) following manufacturer's instructions. Cell nuclei were counterstained with 2 µg/ml Hoechst 33342 (Sigma-Aldrich, St. Louis, MO, USA). Fluorescence images were captured using a DMI4000B confocal microscope (Leica, Deerfield, IL, USA) and analyzed using LAS-AF software (Leica).

### NF-κB/p65 immunofluorescence

GECs were grown on 18 mm glass coverslips and infected with *C. pneumoniae* at a MOI of 5. After three days, the cells were washed, fixed with 4% paraformaldehyde, and permeabilized with 0.1% Triton X-100 in PBS for 40 minutes. Following one wash with PBST (0.05% Tween-20 in PBS) and two washes with PBS, the cells were blocked with 2% BSA in PBS for 30 minutes before hybridization overnight at 4°C with a rabbit monoclonal antibody against NF-κB/p65 (Cell Signaling, catalog #8242). Cells were again washed once with PBST followed by two PBS washes before incubation with Alexa Fluor 546-conjugated goat anti-rabbit IgG (Invitrogen) for 40 minutes. Cell nuclei were counterstained with 2 µg/ml Hoechst 33342 (Sigma-Aldrich). Fluorescence images were captured using a DMI4000B confocal microscope (Leica) and analyzed using LAS-AF software (Leica).

### Quantitative real-time PCR

GECs, HeLa cells and A549 cells were grown in 6-well plates and infected with *C. pneumoniae* at indicated MOI and times as shown in the figure legends. Total RNA was extracted from the infected cells using TRIzol following manufacturer's instructions (Invitrogen) and nucleic acid concentration was determined using a NanoDrop instrument (ThermoScientific Life Sciences, Rockford, IL, USA). cDNAs were synthesized from 2 µg RNA using random hexamers and reagents from the Taqman Reverse Transcription Reagents Kit (Applied Biosystems, Foster City, CA, USA). Using 1:50 of the prepared cDNA, sense and antisense primers, Mx300P (Stratagene, La Jolla, CA, USA) and Brilliant QPCR Master Mix (Stratagene) in a total reaction volume of 25 µL, quantitative PCR was performed with the following thermocycler settings: 95°C for 10 minutes, followed by 40 cycles at 95°C for 30 seconds, 55°C for 1 minute, and 72°C for 30 seconds. *C. pneumoniae* 16S rRNA gene expression was normalized to GAPDH through comparative cycle threshold method. The primers used for *C. pneumoniae* 16S rRNA were: 5′-TTATTAATTGATGGTACAATA-3′ and 5′-ATCTACGGCAGTAGTATAGTT-3′. The primers used for GAPDH were 5′-CTTCTCTGATGAGGCCCAAG-3′ and 5′-GCAGCAAACTGGAAAGGAAG-3′.

### IL-1β ELISA

GECs were seeded into 6-well plates (Corning) and grown to 60% confluency, after which *C. pneumoniae* bacteria were added at a MOI of 5. Supernatants were collected at 0, 1, 2, 3 and 4 days post-infection, centrifuged at 800 x g for 5 minutes to remove cellular debris, and stored at -80°C until ELISA analysis. Measurement of secreted IL-1β proteins was carried out using a human IL-1β ELISA Kit (eBioscience, San Diego, CA, USA) following the manufacturer's instructions.

### Caspase -1 Western blot

A total of 750,000 cells were seeded into each well of 6-well plates (Corning). *C. pneumoniae* was added to the cells at a MOI of 5. Supernatants were collected from uninfected cells (day 0 post-infection) and from infected cells at 1, 2, 3, and 4 days post-infection. All supernatants were centrifuged to remove cellular debris and then subjected to trichloroacetic acid (TCA) precipitation to concentrate the proteins [36]. Briefly, one volume of 100% TCA was added to four volumes of clarified supernatants and incubated for 10 minutes at 4°C. After incubation, samples were centrifuged at 800 x g for 5 minutes and pellets washed twice with 100% cold acetone. Washed pellets were dried by heating at 95°C for 5-10 minutes. Dried pellets were resuspended in Pierce Lane Marker Reducing Sample Buffer (Thermo Fisher Scientific Life Sciences, Rockford, IL, USA), boiled at 95°C for 5 minutes, and loaded onto 12% SDS-polyacrylamide gels. After performance of SDS-PAGE, separated proteins were transferred to polyvinylidene difluoride membranes (PVDF membranes: EMD Millipore, Darmstadt, Germany). The membranes were blocked with 5% BSA (Sigma-Aldrich) for 1 hour at room temperature (RT), and then incubated with 1 mg/ml rabbit anti-human caspase-1 polyclonal antibodies (EMD Millipore) overnight at 4°C. The next day, the membranes were washed and incubated for 1 hour at RT with HRP-conjugated anti-rabbit secondary antibodies diluted 1:10,000 (EMD Millipore). The membranes were washed again, and the proteins were visualized using the ECL Plus Western blot substrate (Amersham Biosciences, Little Chalfont, UK). Chemiluminescent images were acquired using the ChemiDoc XRS+ system (Bio-Rad, Hercules, CA, USA) and analyzed using the ImageJ software.

### NLRP3-deficient GECs

Immortalized GECs stably expressing shRNA against NLRP3 (NCBI accession no: NM_004895, sequence 5′–CCGGGCGTTAGAAACACTTCAAGAACTCGAGTTCTTGAAGTGTTTCTACGCTTTTTG-3′) were generated by transducing the cells with lentiviral particles (Sigma-Aldrich, St. Louis, MO). Transduction was performed according to manufacturer's instructions. Non-target shRNA control was also generated using an irrelevant sequence (NCBI accession no: SHC002; sequence 5′-GGCAACAAGATGAAGAGCACCAACTCGAGTTGGTGCTCTTCATCTTGTTGTTTTT–3′). Briefly, GECs were plated at 35% confluency and grown for 24 hours, after which the corresponding lentiviral transduction particles were added at a MOI of 5 in OPTIMEM medium (Gibco Life Technologies). After 24 hours incubation, stably-infected cells were selected using fresh medium containing 2 μg/ml puromycin (Sigma-Aldrich). The knockdown efficiency was tested by real-time PCR as indicated above. mRNAs were isolated from transduced and non-transduced cells using the Qiagen RNeasy Kit (Qiagen) following the manufacturer's instructions.

### Statistical analysis

When comparing two groups, two-tailed t-test was used. The significance of differences among groups for age was assessed by t-test. Fisher's exact test was used to compare groups regarding sex and education level. Chi square was used to compare the differences in the frequency of different races between the groups. The statistical analysis was performed with Graph Pad Prism v. 5 software. Statistical differences were shown as asterisks, where: ***p<0.001, **p<0.01, *p<0.05.

## SUPPLEMENTAL MATERIAL

Click here for supplemental data file.

All supplemental data for this article are available online at http://www.microbialcell.com/researcharticles/2019a-almeida-da-silva-microbial-cell/.

## Abbreviations:

EB: – elementary body,
GEC: – gingival epithelial cell,
MOI: – multiplicities of infection,
NLR: – NOD-like receptor,
RB: – reticulate body.
