# Supplementary material for: Chlamydia pneumoniae is present in the dental plaque of periodontitis patients and stimulates an inflammatory response in gingival epithelial cells
Source: Microb Cell. 2019 Mar 11;6(4):197–208. doi: 10.15698/mic2019.04.674 (PMC6444558; doi:10.15698/mic2019.04.674)
Supplement: Supplementary file 1 [file mic-06-197-s01.pdf]

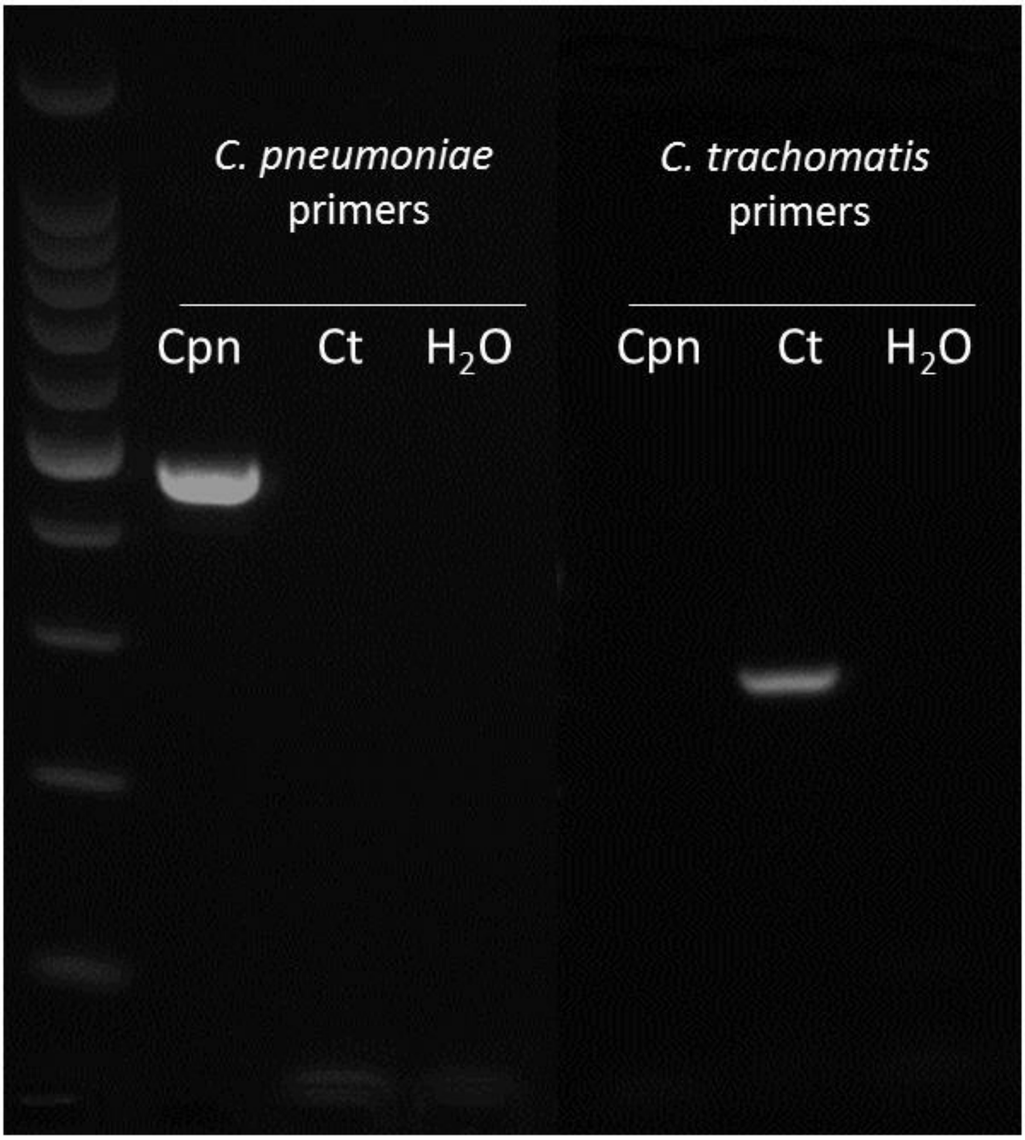

Supplementary FIGURE S1: Primers for the 16S rRNA gene of *C. pneumoniae* and *C. trachomatis* do not show inter-species cross-reactivity. Gel electrophoretogram of PCR products for *C. pneumoniae* and *C. trachomatis* using primers for the 16S rRNA gene of both chlamydial species. Positive controls are genomic DNA from ATCC and negative controls are ultra-pure water. Cpn: *C. pneumoniae*; Ct: *C. trachomatis*; H_2O: water.
